# Nerve growth factor exacerbates allergic lung inflammation and airway remodeling in a rat model of chronic asthma

**DOI:** 10.3892/etm.2013.1284

**Published:** 2013-09-02

**Authors:** YUN-GANG YANG, WEI-MIN TIAN, HAN ZHANG, MIAO LI, YUN-XIAO SHANG

**Affiliations:** Department of Pediatric Pulmonology, Shengjing Hospital of China Medical University, Shenyang, Liaoning 100004, P.R. China

**Keywords:** nerve growth factor, asthma, airway remodeling, T-helper cell type-2 cytokines, matrix metalloproteinase-9

## Abstract

Nerve growth factor (NGF) is critical in the pathogenesis of allergic airway inflammation *in vivo* and induces proliferation of airway smooth muscle cells and matrix metalloproteinase-9 (MMP-9) expression *in vitro*. However, the effects of NGF on chronic pulmonary diseases of allergic origin remain unknown. To investigate the effects of NGF on lung inflammation and airway remodeling, 32 Wistar rats were randomly divided into four groups: control, NGF, ovalbumin (OVA) and anti-rat-β-NGF antibody (anti-NGF). Aerosolized OVA was administered to the rats in the NGF, OVA and anti-NGF groups to generate the asthmatic rat model, and NGF or anti-NGF was administered 3 h prior to OVA inhalation every two days. On day 70, bronchial responsiveness tests, bronchoalveolar lavage (BAL) and cell counting were conducted. The levels of serum OVA-specific immunoglobulin E (IgE) and of T-helper cell type-2 (Th2) cytokines [interleukin (IL)-4 and IL-13] in the BAL fluid were measured by enzyme-linked immunosorbent assay. The expression levels of NGF protein and MMP-9 mRNA, and the activity of MMP-9 in the lungs were detected by western blot analysis, quantitative polymerase chain reaction and gelatin zymography analysis, respectively. Our results showed that NGF significantly increased eosinophilic airway inflammation, persistent airway hyperresponsiveness (AHR), the serum levels of OVA-specific IgE and the levels of Th2 cytokines in the BAL fluid, and also increased the expression levels and activity of MMP-9. However, anti-NGF treatment significantly inhibited eosinophilic airway inflammation, persistent AHR and airway remodeling. The results showed that NGF may have exacerbated the development of airway inflammation, AHR and airway remodeling through a Th2 pathway and by increasing the level of MMP-9 expression. Therefore, anti-NGF is potentially beneficial for preventing and treating patients with asthma.

## Introduction

Asthma is a chronic inflammatory airway disease that is associated with airway remodeling and airway hyperresponsiveness (AHR). Airway remodeling is characterized by the increased deposition of collagen (fibrosis) in the subepithelial basement membrane region and submucosal layers, smooth muscle hypertrophy and hyperplasia, fibroblast hyperplasia, epithelial metaplasia and goblet cell proliferation (1). The poor response to treatment observed in patients with refractory asthma may be a consequence of ongoing airway remodeling that results in fixed airway obstruction (2). Regardless of the advances in understanding the inflammatory and immunological features of asthma, the molecular mechanisms underlying the remodeling changes associated with chronic asthma have not been fully determined.

Nerve growth factor (NGF) is a member of the neurotrophin family of proteins that regulate neuronal development, maintenance and recovery from injury. However, NGF has been implicated in allergic inflammation. High levels of NGF have been detected in the bronchoalveolar lavage fluids (BALF) and serum from asthmatic patients, suggesting that NGF is regulated in the airways and that NGF expression levels are associated with severity of the allergic disease (3,4).

A previous study identified that NGF results in the differentiation of human B cells to plasma cells, modulates chemotaxis and mediates their release by allergic and inflammatory cells. Moreover, NGF enhances chemotaxis and superoxide production by neurotrophils and primes histamine release by human basophils (5).

In addition to neurons and inflammatory cells, NGF affects the contraction, migration, differentiation and proliferation of airway structural cells (6,7). NGF is also synthesized in airway structural cells, such as epithelial cells, pulmonary fibroblasts and smooth muscle cells (8). Several studies have suggested that structural cells may be a target of NGF action in the airways. NGF stimulates the *in vitro* contraction and migration of human pulmonary fibroblasts and their differentiation into myofibroblasts, which induces the proliferation of airway smooth muscle cells through activation of its TrkA receptor and causes matrix metalloproteinase-9 (MMP-9) expression in vascular smooth muscle cells (9,10). These events represent an important step in the airway remodeling process and links NGF to the remodeling mechanism. Therefore, we hypothesized that NGF levels are strongly upregulated and participate in airway remodeling mechanisms. To explore the mechanisms involved in NGF-induced airway remodeling in the airways, we established a rat model of chronic allergic airway inflammation in which the pathological processes of airway remodeling may be demonstrated.

## Materials and methods

### Animals

A total of 32 specific pathogen-free normal female Wistar rats (weight, 120–140 g) were obtained from the Laboratory Animal Research Center in Shengjing Hospital of China Medical University (Shenyang, China). Rats were maintained in a 12 h light:dark cycle with access to food and water *ad libitum*. Initially, the rats were randomly divided into four groups: control, ovalbumin (OVA), NGF and anti-NGF (n=8 per group). All animal experiments were performed in accordance with the National Institute of Health Guide for the Care and Use of Laboratory Animals (8th Edition, 2012). The animal-use protocol has been reviewed and approved by the Institutional Animal Care and Use Committee of the Shengjing Hospital of China Medical University (Shenyang, China).

### OVA sensitization

Sensitization and challenge protocols were performed according to the methods of Li and Shang (11) and Vanacker *et al* (12) with certain modifications as described below. On days 0 and 7, all rats with the exception of those in the control group were actively sensitized with an intraperitoneal (i.p) injection of 1 mg OVA (Grade V; Sigma, St. Louis, MO, USA) and 200 *μ*g aluminum hydroxide (Sigma) in 0.5 ml sterile phosphate-buffered saline (PBS). The OVA-sensitized rats were exposed to 1% aerosolized OVA (1 g OVA in 100 ml sterile PBS in a nebulizer) for 30 min, every two days from day 14 to day 70. As neurotrophic factors are highly conserved in different species, we used exogenous murine NGF (NGF-7S; Alomone Labs, Jerusalem, Israel) and blocked endogenous NGF activity using 100 *μ*g/ml goat anti-rat-β-NGF antibody (R&D Systems, Minneapolis, MN, USA) for our rat model. The NGF and anti-NGF groups were administered an i.p injection of NGF-7S (80 ng/kg) or anti-NGF antibody (4 ml/kg) diluted at 1:1,000 in sterile PBS, respectively, 3 h prior to the OVA aerosol challenge. The administration route, timing and dose of the NGF and anti-NGF treatments were chosen based on a previous study (13). The OVA group received 4 ml/kg PBS 3 h prior to the OVA aerosol challenge. The control group was subjected to the same protocol using sterile PBS.

### Analysis of AHR

Airway reactivity to methacholine (MCH) was assessed *in vivo* 24 h following the last OVA challenge as previously described (14). Rats were anesthetized with 100 mg/kg pentobarbital sodium (i.p), a tracheal cannula was inserted via tracheotomy for mechanical ventilation and a small catheter (22G) was inserted into the external jugular vein for the administration of MCH (Sigma-Aldrich, Beijing, China). The rats were then placed in a sealed whole body plethysmograph and connected to a rodent ventilator (ML-V2; Shanghai Benda Biotechnology Co., Ltd., Shanghai, China). Ambient air was administered with a tidal volume of 8 ml/kg and a frequency of 90 strokes per min. Transducers (ML-AMP II; Shanghai Benda Biotechnology Co.,Ltd.) connected to the ventilatory circuit provided voltage signals of pressure and flow, which were amplified and transmitted to the analog/digital card (National Instruments, Austin, TX, USA) of a microcomputer running the AniRes2005 software (Beijing Bestlab High-Tech Co., Ltd., Beijing, China), which was used to calculate the inspiratory and expiratory resistances of the respiratory system from the digitized pressure and flow signals. Following stabilization of respiratory parameters (10–15 min) rats received MCH (dissolved in 0.9% sodium chloride) intravenously at an initial dose of 0.0625 mg/kg with the dose increasing 2-fold with each injection up to 1 mg/kg to obtain a response curve of lung resistance increase over baseline. Injections were administered at 5-min intervals. The MCH volume was 50 *μ*l, which was administered over 3–4 sec based on the return of resistance curves to the pre-MCH level prior to the next MCH injection. Response was measured as the peak increase above the baseline immediately following MCH administration.

### BAL

Following assessment of airway reactivity, the rats were bled and sacrificed via anesthetic overdose. BAL was performed in the left lungs. The left lungs were washed thrice with 1 ml saline. Lavage fluid was recovered by gentle manual aspiration with a syringe. The retrieved volume, which averaged 75–80% of the instilled saline was immediately centrifuged (10 min, 4°C, 1,000 × g) and the supernatant was stored at −70°C until the levels of IL-4 and IL-13 were measured. The pellet was kept on ice, washed twice with saline and resuspended in 1 ml saline. The total number of leukocytes in the BALF were determined with a Coulter counter (Coulter Electronics Ltd., Harpenden, UK). A differential cell count was performed on Cytospin (Thermo Shandon, Inc,, Pittsburgh, PA, USA) by Wright-Giemsa staining.

### ELISA

After measuring airway reactivity, serum was obtained by lethal cardiac puncture of anesthetized rats and stored at −70°C for measurement of OVA-specific IgE by ELISA.

### Tissue collection

Lung tissues were weighed (total lung weight) and then separated into individual lobes for hydroxyproline analysis, histological analysis, western blotting, quantitative polymerase chain reaction (qPCR) and MMP zymography.

### Lung histopathology

The middle lobes of the right lung were fixed in 4% paraformaldehyde for 18–24 h, embedded in paraffin and then routinely processed. Serial 5-*μ*m tissue sections were stained with hematoxylin and eosin (H&E), Masson’s trichrome and periodic acid-Schiff (PAS) for the assessment of peribronchial inflammation, collagen particles and goblet cells, respectively.

### Morphometric analysis

A minimum of five bronchi (luminal diameter, 150–350 *μ*m) were analyzed per rat for various parameters using a Leica image analysis system (Leica, Cambridge, UK). Using a 10-fold magnification objective, four representative areas were chosen. Subsequently, with a 40-fold magnification corresponding to one microscopic field, hyperplasia of the goblet cells in the epithelial lining was recorded against a score based on the percentage of goblet cells observed in the epithelial cells. The length of the epithelial basement membrane of the bronchus of one area was ≥500 *μ*m. To minimize sampling errors, a 5-point scoring system (grades 0–4) was adopted: grade 0, no goblet cells; grade 1, <25% goblet cells; grade 2, 25–50% goblet cells; grade 3, 50–75% goblet cells; and grade 4, ≥75% goblet cells (15). The mean score of the total epithelial cells in the four areas of one rat were counted. The mean score of hyperplasia of the goblet cells was calculated for 7–8 rats.

Masson’s trichrome-stained tissue sections were used for the assessment of subepithelial fibrosis using the Leica image analysis system. As described previously, three representative areas were chosen using a 10-fold magnification objective. Subsequently, with a 40-fold magnification, epithelial basement membrane areas of ≥250 *μ*m were selected, and the thickness of the epithelial layer and fibrotic area (stained in blue), which were 30 *μ*m beneath the basement membrane of the standardized sampling points, were measured (16). The mean fibrotic area divided by the length of the basement membrane were calculated for 7–8 rats.

### Hydroxyproline analysis

Total collagen content in the fresh lung samples of all rats were determined by hydroxyproline assay. The hydroxyproline content was detected with a commercial hydroxyproline detection kit (Nanjing Jiancheng Bioengineering Institute, Nanjing, China) following the manufacturer’s instructions.

### Western blot analysis

Protein homogenates of lung tissue samples were prepared by rapid homogenization in 10 volumes of lysis buffer (2 mM EDTA, 10 mM EGTA, 0.4% NaF, 20 mM Tris-HCl, 1 mg/ml leupeptin, 1 mg/ml aprotinin and 1 mM Na_3_VO_4_ at pH 7.5). Samples were centrifuged at 12,000 × g for 1 h and the protein concentration of the soluble material was determined using the Coomassie Brilliant Blue G250 (Beyotime Institute of Biotechnology, Jiangsu, China) binding method (17). Proteins (10 *μ*g) from each sample were loaded onto an 8% sodium dodecyl sulfate-polyacrylamide gel. Electroblotted proteins were transferred from the gel to nitro-cellulose membranes, which were incubated with 1:1,000 goat anti-NGF. The NGF band (140 kD) was visualized using an enhanced chemiluminescence kit (Santa Cruz Biotechnology, Inc., Santa Cruz, CA, USA). Integrated density values (IDV) were analyzed using a computerized image analysis system (Fluor Chen 2.0; Bio-Rad, Hercules, CA, USA) and normalized to those of β-actin.

### qPCR

Rat lung tissues were dissected and stored in TRIzol reagent (Invitrogen Life Technologies, Carlsbad, CA, USA). The levels of MMP-9 mRNA were determined using the ABI PRISM 7500 Real-Time PCR system (Applied Biosystems, Foster City, CA, USA). The RNA was extracted using TRIzol reagent in accordance with the manufacturer’s instructions. RNA purity was determined and cDNA synthesis was conducted using a SYBR^®^ PrimeScript™ RT-PCR kit (Takara Biotechnology, (Dalian) Co., Ltd., Dalian, China). The volume of rat MMP-9 mRNA was determined by qPCR. The primers for MMP-9 were 5′-CCCACTTACTTTGGAAACG-3′ (forward) and 5′-GAAGATGAATGGAAATACGC-3′ (reverse), and those for GAPDH were 5′-GCAAGTTCAACGGCACA-3′ (forward) and 5′-CATTTGATGTTAGCGGGAT-3′ (reverse) [Takara Biotechnology (Dalian) Co., Ltd]. Gene expression levels of MMP-9 were analyzed by the 2^−^^ΔΔ^^CT^ method (18).

### Zymography

Total MMPs were extracted from a similar section of each lung tissue and analyzed by gelatin zymography (Genmed Scientifics Inc., Arlington, MA, USA) according to the manufacturer’s instructions. The resulting bands on the zymograph were analyzed by densitometry using a GS-710 densitometer (Bio-Rad Laboratories, Richmond, CA, USA) and Quantity-One software (Bio-Rad). The mean ± standard error density of each MMP was plotted in a graph and expressed as the relative ratio of the values in the control group, which were expressed as one.

### Statistical analysis

Data are expressed as the mean ± standard error of the mean for each group. One-way analysis of variance (ANOVA) followed by the Bonferroni post hoc test was used to compare group differences, whereas two-way ANOVA was used to assess differences in airway resistance. P<0.05 was considered to indicate a statistically significant difference.

## Results

### Airway resistance measurement

**Figure 1.**
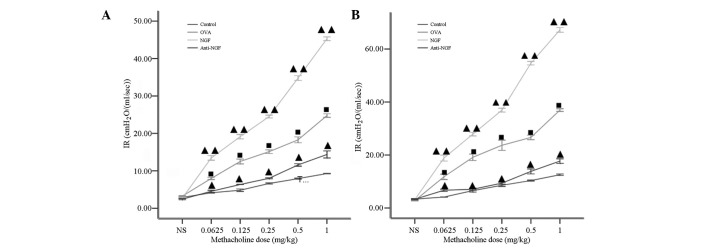
Airway (A) inspiratory and (B) expiratory resistance in response to varied doses of MCH in the four groups were analyzed using an animal lung function apparatus. Dose-response curves showed that the airway response to MCH was significantly increased in the OVA and NGF groups compared with that of the control group (P<0.01). Treatment of OVA-sensitized rats with anti-NGF prevented this increase in airway reactivity (P<0.01). ▪P<0.01 compared with the control group, ^▴^P<0.01 and ^▴▴^P<0.05 compared with the OVA group. MCH, methacholine; OVA, ovalbumin; NGF, nerve growth factor; IR, inspiratory resistance; ER, expiratory resistance.

All experimental rats exhibited dose-dependent augmentation of inspiratory and expiratory resistance in response to increasing doses of intravenously administered MCH. No statistically significant differences in baseline inspiratory and expiratory resistance values among all experimental groups was observed. The airway response to MCH significantly increased in the OVA and NGF groups compared with that of the control group (P<0.01). Treatment of OVA-sensitized rats with anti-NGF prior to the OVA challenge prevented the increase in airway reactivity, which was reflected by a shift in the dose-response curves (Fig. 1) and significantly decreased reactivity compared with that of the OVA group (P<0.01).

### Airway inflammation

To investigate the function of NGF in antigen-induced inflammatory infiltration in the airways, we examined the effects of NGF and anti-NGF antibodies in the asthmatic rat model. As shown in Table I, the number of total cells, eosinophils, macrophages, lymphocytes and neutrophils increased in the BALF of the untreated OVA-sensitized and NGF-treated rats compared with those in the PBS-treated rats (controls) (Table I). By contrast, anti-NGF treatment significantly inhibited the increase in the number of total leukocytes, eosinophils and lymphocytes in the BALF following antigen inhalation.

### Goblet cell hyperplasia

**Figure 2.**
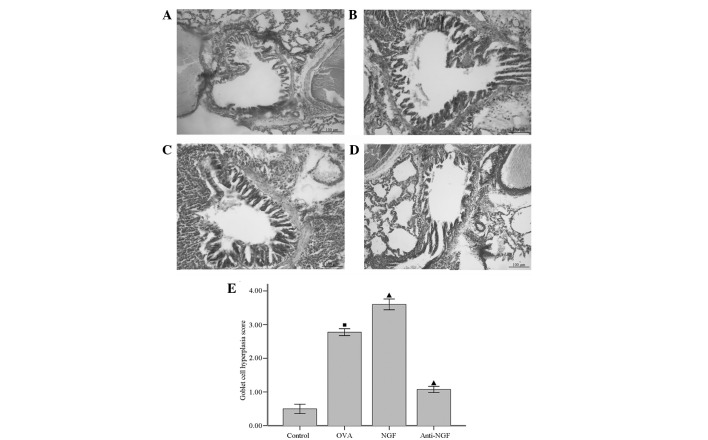
Effects of NGF on antigen-induced goblet cell hyperplasia in sensitized rats 24 h following the final antigen challenge (magnification, ×100). (A–D) Lung sections stained with periodic acid-Schiff. Scale bar: 100 *μ*m. (A) PBS-treated, (B) OVA-treated, (C) NGF-treated, and (D) anti-NGF treated rats. (E) Goblet cell hyperplasia was scored by five grades (0–4). Values are expressed as the means ± standard error of the mean of 8 rats in each treatment group. ^▪^P<0.01 compared with the control group and ^▴^P<0.01 compared with the OVA group. NGF, nerve growth factor; PBS, phosphate-buffered saline; OVA, ovalbumin.

To evaluate the effects of NGF on antigen-induced goblet cell hyperplasia in the airway epithelium, which is a cardinal feature of bronchial asthma, lung sections were stained with PAS for detection (Fig. 2). Goblet cell hyperplasia was then quantitatively estimated in terms of grade as described in Materials and methods (Fig. 2E). As shown in Fig. 2A, histological analyses of lungs from the PBS-treated rats showed normal lung histology. However, the number of goblet cells in the epithelium greatly increased following repeated antigen challenge (Fig. 2B), and goblet cells in the epithelium showed hypertrophic features. Antigen-induced goblet cell hyperplasia was greatly increased by NGF administration (Fig. 2C), but significantly reduced by anti-NGF treatment (Fig. 2D).

### Allergen-induced airway remodeling

**Figure 3.**
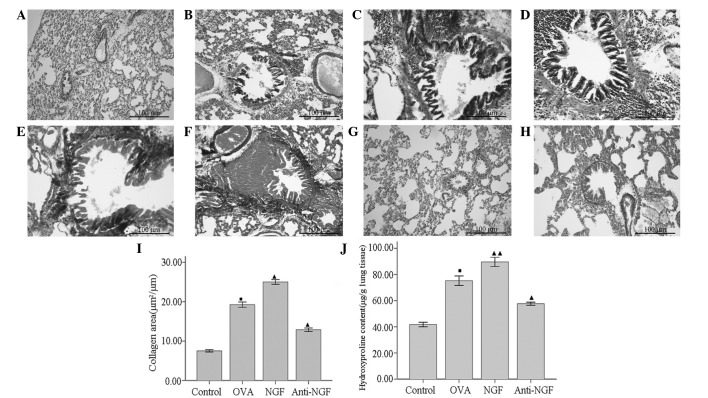
Histological analysis of lung sections stained with (A–D) hematoxylin and eosin and (E–H) Masson’s trichrome 24 h following the final antigen challenge in rats (magnification, ×100). (A and G) PBS-treated, (B and H) anti-NGF-treated, (C and E) OVA-treated and (D and F) NGF-treated rats. ^▪^P<0.01 compared with that of the control group, ^▴^P<0.01 and ^▴▴^P<0.05 compared with that of the OVA group. PBS, phosphate-buffered saline; NGF, nerve growth factor; OVA, ovalbumin.

To determine whether NGF is involved in the development of airway remodeling, we evaluated the peribronchial cellular infiltration, airway smooth muscle thickness and lung collagen content in all experimental rats. Representative sections of each group were stained with either H&E (Fig. 3A–D) or Masson’s trichrome (Fig. 3E–H). As shown in Fig. 3A, no inflammation, mucosal edema and epithelial lesions were observed in the control group. Moderate inflammation, mucosal edema and epithelial lesions were observed in the anti-NGF group (Fig. 3B), whereas sensitization in the OVA group was associated with predominantly moderate to severe inflammation, mucosal edema and epithelial lesions (Fig. 3C). In particular, only NGF-treated rats developed severe inflammation, mucosal edema and epithelial lesions, which included interstitial infiltrates and large lymphoid aggregates (Fig. 3D). Peribronchial fibrosis (Fig. 3I) and lung collagen content (Fig. 3J) were quantitatively estimated. Exposure to OVA increased the peribronchial trichrome-stained area and lung collagen levels in the OVA (Fig. 3E) and NGF groups (Fig. 3F) when compared with those in the control group (Fig. 3G). Anti-NGF-treated rats showed reduced peribronchial collagen staining (Fig. 3H) compared with that in the OVA-treated rats. Lung collagen levels in the anti-NGF-treated rats were significantly reduced (57.6±1.19 *μ*g collagen/g lung tissue) compared with those in the OVA-treated rats (75.23±3.64 *μ*g collagen/g lung tissue) (P<0.01).

### Cytokine response

We analyzed the concentrations of Th2-associated cytokines (IL-4 and IL-13) in the BALF. As shown in Table II, the IL-4 and IL-13 levels were higher in the BALF of the untreated OVA-sensitized and NGF-treated rats compared with those of the PBS-treated rats (controls). Anti-NGF-treated rats showed reduced levels of IL-4 and IL-13 compared with those of the untreated OVA-sensitized rats.

### Serum levels of OVA-specific IgE

To confirm that the presence of the OVA antigen resulted in immunological sensitization, OVA-specific IgE serum levels were measured. Serum IgE levels were increased in all OVA-sensitized groups compared with those of the control group. However, serum IgE levels were reduced in the anti-NGF group compared with those of the OVA group (Table II).

### Western blot analysis

**Figure 4.**
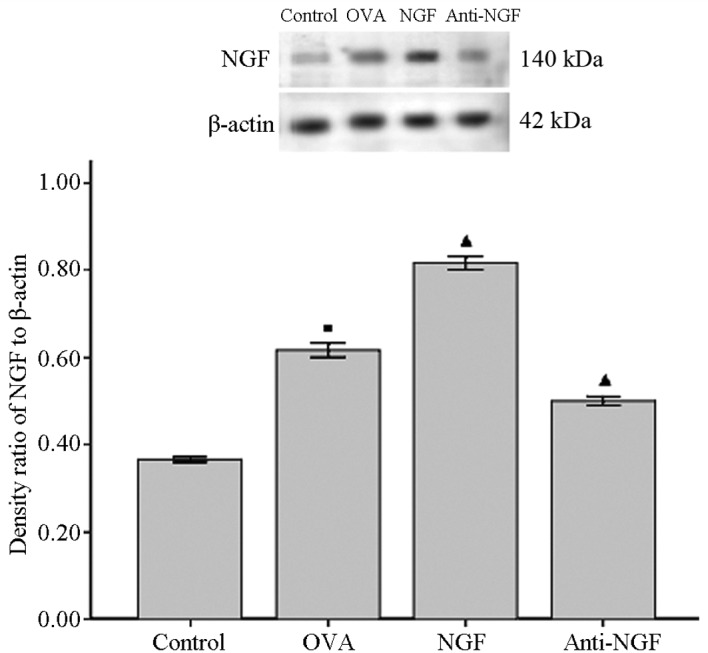
Western blot analysis of NGF in the lungs of the four treatment groups. Treatment with NGF increased the levels of NGF protein. Data are expressed as the mean ± standard error of the mean (n=8 per group). ^▪^P<0.01 compared with the control group and ^▴^P<0.01 compared with the OVA group. NGF, nerve growth factor; OVA, ovalbumin.

NGF protein levels in all groups were detected by western blotting. In the lung tissues of the control group, NGF was expressed at low levels, but these levels were markedly increased in the OVA and NGF groups. However, anti-NGF treatment markedly decreased the NGF upregulation caused by OVA and NGF (Fig. 4). The mean IDV ratio of NGF to β-actin was 0.36±0.01 in the control group, 0.62±0.02 in the OVA group, 0.50±0.02 in the anti-NGF group and 0.81±0.02 in the NGF group (Fig. 4).

### qPCR

**Figure 5.**
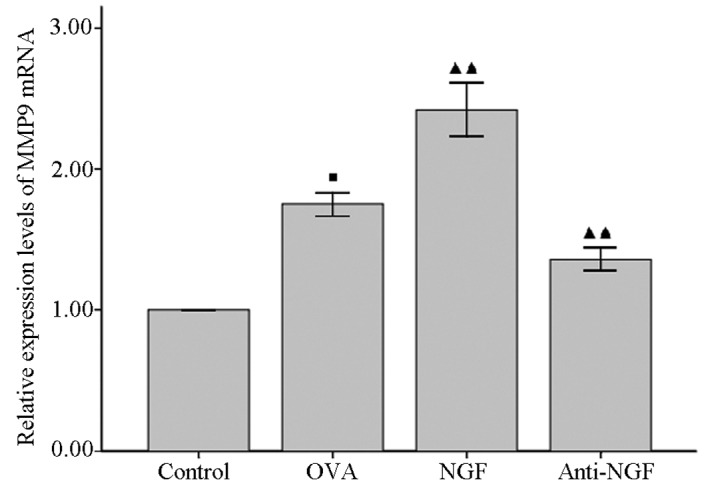
qPCR analysis of the levels of MMP-9 in the lungs of the four treatment groups. Treatment with NGF increased the expression levels of MMP-9 mRNA. Data are expressed as the mean ± standard error of the mean (n=8 per group). ^▪^P<0.01 compared with the control group and ^▴▴^P<0.05 compared with the OVA group. qPCR, quantitative polymerase chain reaction; MMP, matrix metalloproteinase; NGF, nerve growth factor; OVA, ovalbumin.

qPCR was used to examine the expression levels of MMP-9 mRNA in the pulmonary tissues of all groups. MMP-9 mRNA expression levels were significantly higher in the OVA and NGF groups than in the control group, but anti-NGF treatment significantly decreased MMP-9 mRNA expression levels in the OVA-sensitized rats (Fig. 5).

### Lung zymography

**Figure 6.**
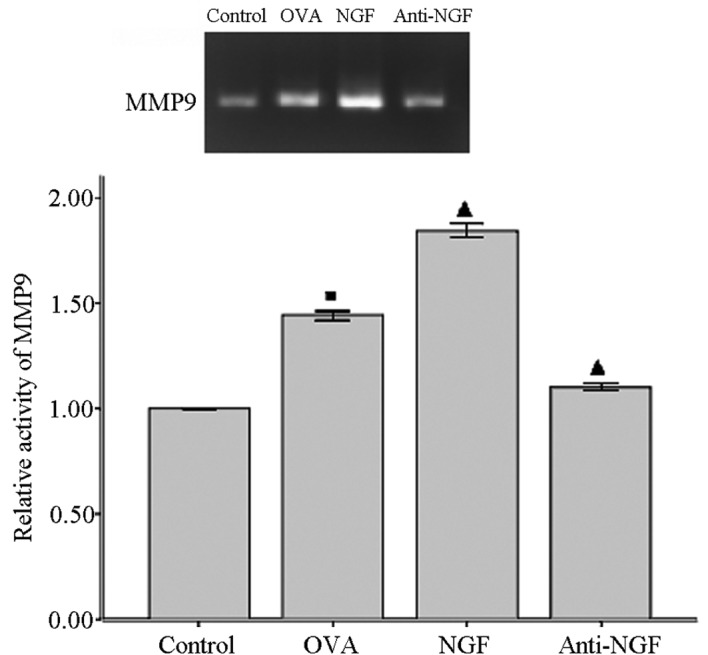
Gelatin zymography for MMP-9 activity in the lungs of the four treatment groups. Treatment with NGF increased MMP-9 activity in the lung tissues. Data are expressed as the mean ± standard error of the mean (n=8 per group). ^▪^P<0.01 compared with the control group and ^▴^P<0.01 compared with the OVA group. MMP, matrix metalloproteinase; NGF, nerve growth factor; OVA, ovalbumin.

Gelatin zymography of the lung tissues from the rats in all experimental groups was used to demonstrate changes in the activity of MMP-9. Densitometric analysis of the zymographs demonstrated that MMP-9 activity in the lung tissues of all OVA-sensitized rats was significantly increased compared with that of the controls (Fig. 6). By contrast, a significant reduction in MMP-9 activity in the lung tissues of the anti-NGF-treated rats compared with that in the OVA-treated rats was observed.

## Discussion

In the present study, we evaluated the effects of NGF on an experimental rat model of chronic allergic lung inflammation in rats. Our results showed that NGF exacerbated allergic inflammation, airway responsiveness and airway remodeling in sensitized rats, characterized by a significant increase in the number of inflammatory cells in the BALF and airway walls, AHR and in the volume proportion of collagen fibers. Moreover, NGF-induced changes were shown to be mediated by an increase in the release of IL-4 and IL-13 by inflammatory cells. Notably, anti-NGF treatment decreased all studied parameters of the OVA-sensitized rats. This indicates that anti-NGF administration decreased airway inflammation, remodeling and AHR in OVA-sensitized rats.

The functions of NGF in allergic airway inflammation have been investigated using NGF transgenic, NGF-treated or anti-NGF-treated mice. Numerous findings have indicated that NGF may influence developmental differentiation, chemo-taxis and mediate the release of inflammatory cells (19,20). However, the involvement of NGF in antigen-induced airway eosinophilic inflammation and in the pathogenesis of allergen-induced airway remodeling have not been fully investigated. Kerzel *et al* demonstrated that in a model of experimental asthma, transgenic mice, which constitutively overexpressed NGF in the lung epithelial cells, recruited significantly more eosinophils following allergic sensitization than the wild-type animals (21). By contrast, p75NTR-deficient mice exhibited a significant reduction in eosinophilic infiltration (22) and similar effects were found following the inactivation of NGF by the intranasal application of an anti-NGF antibody during allergic sensitization (23). These findings clearly demonstrated that NGF in the airways may have detrimental effects on asthma. However, the exogenous administration of NGF or NGF-neutralizing antibodies did not modify IgE and eosinophil parameters; whereas in control rats, NGF administration did not induce an increase in IgE or eosinophils in the BALF and lungs (24).

Therefore, prior to evaluating the effects of NGF, we first examined whether NGF levels increased following repeated antigen challenge in our chronic asthma model. The repeated antigen challenge resulted in increased levels of NGF protein in the lung tissues and of OVA-specific IgE in the serum compared with the respective levels in the control rats. As expected, anti-NGF administration markedly inhibited the increase of NGF protein levels and serum levels of OVA-specific IgE; whereas NGF treatment significantly promoted the increase. Mast cells are resident tissue cells, and in allergic diseases they are critical effector cells as they are the main contributors to immediate hypersensitivity reactions when activated through IgE and specific antigens. Mast cells predominantly depend on NGF for homing, survival and differentiation. NGF is a chemoattractant for mast cells and acts as a cofactor together with the stem cell factor to prevent apoptosis (25). NGF and the combination of NGF and stem cell factor increase or induce the expression of typical mast cell markers, such as IgE-receptor type I, chymase or mast-cell specific tryptase.

In allergic inflammatory responses, a number of cytokines and chemokines are released from various cell types. In bronchial asthma, CD4^+^ T cells produce and secrete a large quantity of Th2 cytokines, such as IL-4, IL-5 and IL-13, and these cytokines promote allergic airway inflammation (26). In the present study, the administration of NGF significantly exacerbated airway eosinophilic inflammation, increased the total inflammatory cell number and the production of IL-13 and IL-4 in the BALF, as well as goblet cell hyperplasia in the epithelium whereas treatment with anti-NGF clearly reduced these factors. The observed changes in pulmonary inflammation and mucus production possibly resulted from concomitant changes in the levels of Th2-type cytokines, IL-4 and IL-13 in the BALF (27). Moreover, administration of NGF aggravated AHR. The precise mechanisms underlying allergen-induced AHR remain unclear; however, recent studies using IL-4-gene-knockout mice and IL-13-gene-knockout mice have demonstrated that AHR is dependent on Th2 cytokines (28,29). In conclusion, these observations indicate that Th2 cytokines are important in the development of allergen-induced AHR. Therefore, increased Th2 cytokine production in the BALF may subsequently result in aggravated AHR, which was observed in NGF treatment.

Notably, regardless of exacerbated lung inflammation and AHR, the administration of NGF further increased collagen deposition in the airway walls. We suggest that these changes may have been mediated by an increase in MMP-9 activity in the lung tissues, which was observed in our animals following NGF administration. MMP-9 belongs to a family of extracellular proteases that are responsible for the degradation of the extracellular matrix during tissue remodeling (26). Levels of MMP-9 (gelatinase B) are significantly increased in the BALF of OVA-sensitized BALB/c mice, blood and sputum of patients with allergic asthma (30,31). Studies of MMP-9-deficient mice demonstrated that when challenged with an allergen, mice showed relatively less peribronchial fibrosis and total lung collagen compared with that of the wild-type mice (32). In terms of the potential effects of MMP-9 on airway inflammation in asthma, *in vitro* studies have shown that MMP-9 may be important in mediating the migration of eosinophils across the basement membrane of blood vessels (33). Moreover, *in vitro* studies have identified that NGF may induce MMP-9 expression in vascular smooth muscle cells and human bronchial smooth muscle cells, and induce the proliferation of airway smooth muscle cells through activation of its TrkA receptor (9,34). We speculate that the increase in collagen deposition in the airway walls and the enhanced smooth muscle thickness induced by NGF are possibly mediated by the increased expression of MMP-9.

In conclusion, our results suggest that NGF exacerbated the effects of OVA sensitization (airway inflammation, remodeling and hyperresponsiveness) via Th2 and MMP-9 pathways. However, the extent to which the results obtained in our rat model of allergic inflammation may be transposed to patients with asthma remain unclear.

NGF has been used as a therapeutic agent for Alzheimer’s disease, peripheral neuropathies, amyotrophic lateral sclerosis and human corneal and skin ulcers (35,36). No deleterious effects of NGF have been confirmed. However, the exacerbation of allergic chronic lung inflammation observed in the present study suggests that NGF treatment may exhibit deleterious effects in individuals with allergic conditions, such as asthma, whereas anti-NGF treatment is potentially beneficial in the prevention and treatment of asthma.

## Figures and Tables

**Table I.** Total white blood cells and cellular composition in the BALF.

Group	Total cells (×10^6^)	Cellular Composition (%)			
		Macrophages	Eosinophils	Neutrophils	Lymphocytes
Control	3.64±0.20	94.06±0.45	1.45±0.09	1.33±0.48	3.52±1.31
OVA	12.23±0.43^a^	57.75±0.63^a^	4.58±0.19^a^	32.05±0.79^a^	5.61±0.85
NGF	17.18±0.44^b^	50.13±0.69^b^	5.42±0.15^c^	37.75±0.37^b^	6.68±0.58^a^
Anti-NGF	5.43±0.36^b^	81.25±0.62^b^	2.96±0.23^b^	11.37±0.56^b^	4.41±0.63

Values are expressed as the mean ± standard error of the mean of 8 rats in each treatment group.

a P<0.01 compared with the control group,

b P<0.01 and

c P<0.05 compared with the OVA group. BALF, bronchoalveolar lavage fluid; OVA, ovalbumin; NGF, nerve growth factor.

**Table II.** Effects of NGF on the levels of IL-4 and IL-13 in the BALF and serum levels of OVA-specific IgE.

Group	BALF (pg/ml)		Serum (*μ*g/ml) OVA-specific IgE
	IL-4	IL-13	
Control	21.25±2.78	71.50±3.79	-
OVA	66.00±2.27^a^	132.50±4.64^a^	11.15±0.43^a^
NGF	82.50±4.05^b^	149.75±3.12^b^	14.98±0.73^c^
Anti-NGF	51.00±3.02^b^	90.75±2.32^c^	5.63±0.46^c^

Treatment with NGF increased the levels of the Th2 cytokines IL-4 and IL-13 in the BALF and the total serum levels of IgE in the rat model of chronic asthma. However, administration of anti-NGF reduced the levels of IL-4 and -13 in the BALF and total serum IgE. Values are expressed as the mean ± standard error of the mean of 8 rats in each treatment group.

a P<0.01 compared with the control group,

b P<0.05 and

c P<0.01 compared with the OVA group. NGF, nerve growth factor; IL, interleukin; BALF, bronchoalveolar lavage fluid; IgE, immunoglobulin E; OVA, ovalbumin.
